# Wavelength-time-division multiplexed fiber-optic sensor array for wide-field photoacoustic microscopy

**DOI:** 10.1016/j.pacs.2025.100725

**Published:** 2025-04-18

**Authors:** Wei Li, Xiaoxuan Zhong, Jie Huang, Xue Bai, Yizhi Liang, Linghao Cheng, Long Jin, Hao-Cheng Tang, Yinyan Lai, Bai-Ou Guan

**Affiliations:** a Guangdong Provincial Key Laboratory of Optical Fiber Sensing and Communications, Institute of Photonics Technology, College of Physics & Optoelectronic Engineering, Jinan University, Guangzhou, China; b MOE Key Laboratory of Laser Life Science, Guangdong Key Laboratory of Laser Life Science, School of Optoelectronic Science & Engineering, South China Normal University, Guangzhou, China; c Department of Otorhinolaryngology, Nanfang Hospital, Southern Medical University, Guangzhou, China; d Otorhinolaryngology Hospital, The First Affiliated Hospital of Sun Yat-sen University, Sun Yat-sen University, Guangzhou, China

**Keywords:** Photoacoustic microscopy, Fiber-optic sensors, Wavelength-time-division multiplexing, Wide-field imaging, Hemodynamic monitoring

## Abstract

Photoacoustic microscopy (PAM) faces a fundamental trade-off between detection sensitivity and field of view (FOV). While optical ultrasound sensors offer high-sensitivity unfocused detection, implementing multichannel detection remains challenging. Here, we present a wavelength-time-division multiplexed (WTDM) fiber-optic sensor array that assigns distinct wavelengths to individual sensors and employs varying-length delay fibers for temporal separation, enabling efficient multichannel detection through a single photodetector. Using a 4-element sensor array, we achieved an expanded FOV of 5 × 8 mm² while maintaining high temporal resolution (160 kHz A-line rate, 0.25 Hz frame rate) and microscopic spatial resolution (10.7 μm). The system's capabilities were validated through comparative monitoring of cerebral and intestinal hemodynamics in mice during hypercapnia challenge, revealing distinct temporal patterns with notably delayed recovery in cerebral vascular response compared to intestinal vasculature. This WTDM approach establishes a promising platform for large-field, high-speed photoacoustic imaging in biomedical applications.

## 1 Introduction

Photoacoustic microscopy (PAM) has emerged as a powerful biomedical imaging technology that combines optical excitation with acoustic detection to achieve high-resolution visualization of biological structures and functions [1], [2]. This hybrid imaging modality offers unique advantages in capturing both structural and functional information of biological tissues, particularly in applications requiring real-time monitoring of dynamic physiological processes. Recent technological advances in PAM have primarily focused on improving imaging speed and field of view (FOV). Various laser scanning mechanisms have been developed, including voice-coil scanner [3], galvanometer scanner [4], [5], [6], micro-electro-mechanical system (MEMS) scanners [7], [8], [9], [10] including double spiral resonant scanning [11], slider-crank scanners [12], [13] and polygon-mirror scanners [14], [15], [16]. Parallel excitation strategies, such as multiple laser beams generated by microlens arrays [17], [18], [19], [20] or beam splitters [21], have further enhanced imaging speed. However, the detection aspect of PAM systems continues to present significant challenges, particularly in achieving simultaneous high-speed imaging over large FOVs while maintaining high sensitivity.

Conventional piezoelectric transducers (PZT) have been widely adopted in PAM systems [16], [22], [23], while transparent ultrasound transducers (TUT) represent an innovative advancement [24], [25], [26] that effectively addresses the optical-acoustic confocal alignment challenges and enables more compact system designs. Both technologies, however, face certain trade-offs in simultaneously achieving high detection sensitivity and large FOV. When implementing spherically or cylindrically focused configurations to enhance sensitivity,the FOV is typically in below 1 mm^2^ mechanical scanning becomes necessary to cover larger FOVs. This scanning approach, while effective, introduces additional complexity and potential motion artifacts, particularly challenging for applications requiring high system stability and real-time imaging capability. Recent developments in non-focused TUTs have shown promising results in system miniaturization, though balancing sensitivity and FOV remains an ongoing challenge in the field.

Optical ultrasound sensors have emerged as a compelling alternative, offering superior performance through resonance-enhanced detection of acoustic-induced phase modulations [27], [28]. These sensors excel in their ability to combine miniaturized footprints with broad detection bandwidths and high sensitivity while maintaining large FOV (up to 40 mm^2^) capabilities using optical scanning. The versatility of optical sensing platforms is demonstrated through various implementations, including Fabry–Pérot interferometers [29], [30], [31], [32], [33], [34], micro-ring resonators [35], [36], [37], π-phase shifted fiber Bragg gratings [38], [39], [40], [41], and laser cavities [42], [43]. Furthermore, their successful integration on diverse material platforms—ranging from silica and silicon to polymeric substrates [44], [45], [46],—has enabled breakthrough applications in wearable devices [47] and endoscopic imaging systems [43], [48], [49], [50]. However, the implementation of optical sensor arrays for parallel ultrasound detection and FOV expansion presents significant technical challenges. Current wavelength-division multiplexing (WDM) approaches, utilizing either wideband pulsed interferometry [51] or digital optical frequency combs with densely spaced laser frequencies for resonator array interrogation [52], exhibit an inherent sensitivity degradation as the sensor count increases, fundamentally limiting array scalability. While alternative configurations using multiple Fabry-Perot cavity sensors can achieve excellent performance, they require individual photodetectors, acquisition channel and interferometer interrogation systems for each sensor element, resulting in increased system complexity and cost that makes them less practical for large-field photoacoustic microscopy applications [53], [54], [55].

**Fig. 1:**
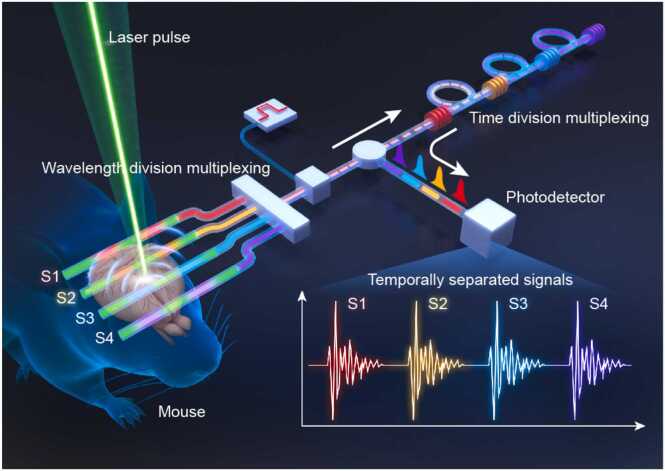
Schematic illustration of the wavelength-time-division multiplexed fiber laser ultrasound sensor array for wide-field photoacoustic microscopy.

To overcome these limitations, we developed a wavelength-time-division multiplexed (WTDM) fiber laser ultrasound sensor array. This novel hybrid multiplexing strategy synergistically combines wavelength-division multiplexing with temporal encoding, enabling efficient parallel ultrasound detection through a single photodetector while maintaining optimal sensitivity across all channels. We demonstrated the system's capabilities using a 4-element fiber ultrasonic sensor array (Fig. 1), achieving a significant FOV expansion (5 × 8 mm²) with pure optical scanning while preserving high temporal resolution (160 kHz A-line rate, 0.25 Hz frame rate) and microscopic spatial resolution (10.7 μm). The system's biological imaging capabilities were validated through comparative studies of cerebral and intestinal hemodynamics, revealing distinctive temporal patterns characterized by delayed response and recovery in cerebral vessels compared to intestinal vasculature. The integration of our compact fiber optic sensor array with the efficient multiplexing strategy establishes a versatile platform for developing portable, wearable, and handheld PAM systems. This technological breakthrough holds significant potential for both preclinical research and clinical diagnostics, enabling real-time, large-field imaging across multiple anatomical regions simultaneously.

## 2 WTDM sensor array implementation

**Fig. 2:**
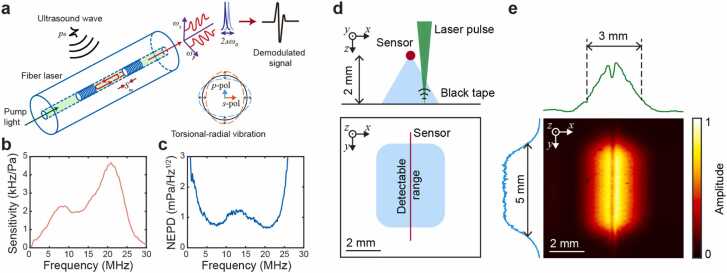
Working principle and comprehensive characterization of an optical ultrasound sensor. (a) Schematic diagram illustrating the dual-polarization fiber laser sensor design, showing ultrasound-induced torsional-radial vibration and the corresponding p- and s-polarization components. (c) Frequency-dependent sensitivity spectrum. (d) Noise equivalent pressure density (NEPD) spectrum. (d) Experimental setup for spatial sensitivity characterization. (e) Spatial sensitivity distribution analysis. Top and left panels: normalized one-dimensional sensitivity profiles along x- and y-axes. Center panel: two-dimensional sensitivity map with normalized amplitude represented in pseudo-color scale.

The ultrasound detection utilizes our previously reported dual-polarization single-longitudinal fiber laser sensor [43], [47]. As shown in Fig. 2(a), the sensor consists of a 5-mm cavity formed by two highly reflective Bragg gratings inscribed in rare-earth-doped fibers (EY305, Coractive). This compact configuration ensures single-longitudinal mode operation with kilohertz-level linewidth. The fiber's intrinsic birefringence (10^−6^) generates two orthogonally polarized modes with distinct frequencies (*ω*_s_ and *ω*_p_), which undergo heterodyne beating through a 45°-oriented polarizer. The resultant radio-frequency beat signal (*ω*_b_=|*ω*_s_-*ω*_p_|) is detected by a high-speed photodetector (DSC50S, Discovery Semiconductors). Incident ultrasonic waves (*p*_0_) induce torsional-radial vibration in the fiber, resulting in equal but opposite frequency modulations (± Δ*ω*_0_) of the orthogonal modes, effectively doubling the beat signal frequency shift (2Δ*ω*_0_) and enhancing detection sensitivity. The sensor's frequency response spectrum, characterized using a calibrated needle hydrophone (NH0200, Precision Acoustics Ltd.), exhibits a characteristic resonance peak at 22 MHz corresponding to the fiber's torsional-radial vibrational mode (Fig. 2b), while the noise equivalent pressure density (NEPD) measurement demonstrates an average sensitivity of 1 mPa/Hz^1/2^ across 5–25 MHz (Fig. 2c).

The FOV was characterized by raster scanning a pulsed laser beam on a black tape target positioned 2 mm beneath the sensor (Fig. 2d). The measured sensitivity field exhibits an elliptical profile (Fig. 2e) with an effective detection area of 3 mm × 5 mm at the −6 dB contour. The sensitivity distribution along the y-axis follows the intracavity laser-light intensity profile, while the x-axis distribution reflects the directional characteristics of torsional-radial vibrational modes, showing maximum response at normal incidence and decreasing sensitivity at oblique angles. A minor dip in the x-axis profile (approximately 200 μm in width) due to fiber shadowing can be observed. This minimal impact can be further reduced through strategic sensor positioning relative to the imaging plane. Our in vivo imaging result in Fig. 5 shows that when the focal plane is positioned 4 mm from the sensor array, the shadow effect becomes negligible. While this increased working distance theoretically results in a *d*^−1/2^ dependent signal attenuation (approximately 3 dB SNR decrease from 2 mm to 4 mm distance) [56], our sensor's high intrinsic sensitivity ensures maintained image quality without significant degradation. Further optimization can be achieved through higher system NA implementation, use of index-matching coupling media, and strategic sensor placement optimization. The system's lateral resolution, determined through edge spread function (ESF) and line spread function (LSF) analysis of a sharp blade edge, achieves 10.7 μm.

**Fig. 3:**
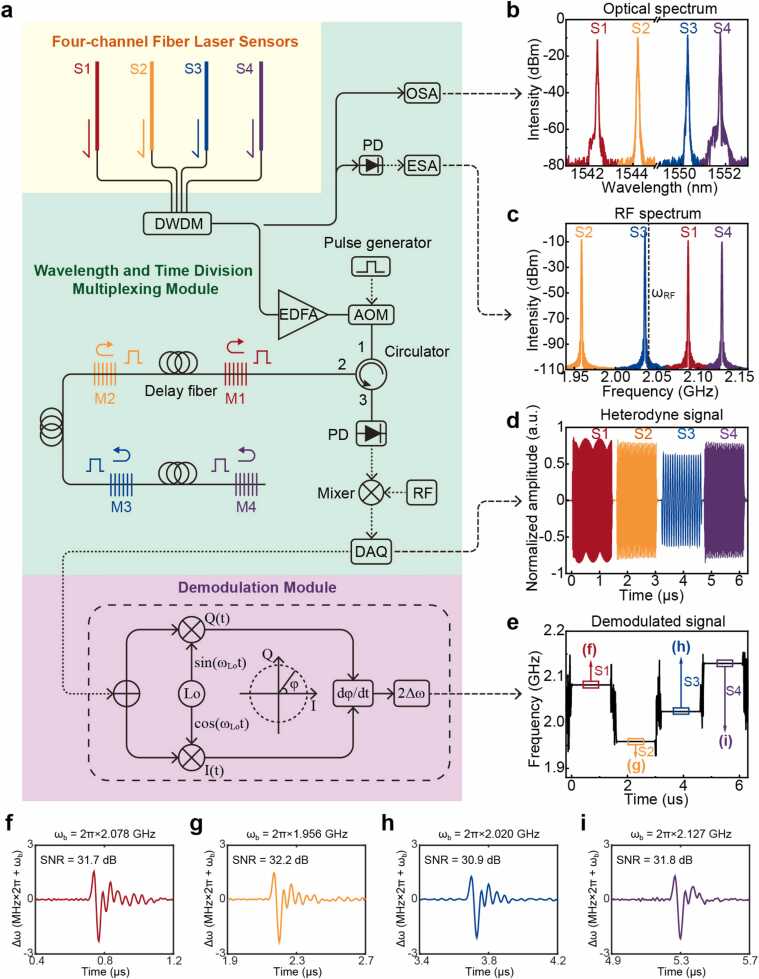
Implementation of the multiplexed sensor array. (a) Schematic diagram of the multichannel ultrasound detection system. Key components include DWDM: dense wavelength division multiplexer; EDFA: Erbium-doped fiber amplifier; OSA: optical spectrum analyzer; ESA: electrical spectrum analyzer; AOM: acousto-optic modulator; PD: photodetector; RF: radio frequency; DAQ: data acquisition card; M1–4: mirror composed of Bragg grating. (b) Optical spectra of the four sensing channels showing distinct wavelength separation. (c) Radio frequency spectra demonstrating the carrier frequencies of individual sensors. (d) Time-sequenced waveforms of the downconverted signals acquired through temporal gating. (e) Demodulated ultrasonic signals from the four sensors showing their respective temporal positions and frequency characteristics. (f–i) The result of simultaneous detection of the same ultrasonic signal by four sensors.

Fig. 3 presents our wavelength-time-division multiplexing (WTDM) ultrasound array system, comprising three core modules: a four-channel fiber laser sensor array, a hybrid WTDM unit, and an IQ demodulation module. The sensor array, as depicted in Fig. 3a, integrates four fiber laser sensors (S1–S4). Leveraging the mature dense wavelength-division multiplexing (DWDM) technology from optical telecommunications, each sensor operates at a standardized wavelength channel in the C-band (1542.94 nm, 1544.53 nm, 1550.92 nm, and 1551.76 nm, respectively). This telecommunications-grade DWDM implementation ensures precise wavelength control and optimal spectral separation between channels, while maintaining high system reliability and cost-effectiveness. The optical spectrum (Fig. 3b) demonstrates well-defined spectral separation between channels, with each sensor's output individually color-coded for clarity.

The WTDM unit implements a two-stage multiplexing strategy. Initially, sensor outputs undergo wavelength-division multiplexing through a DWDM followed by erbiurm-doped fiber amplifier (EDFA) to achieve uniform 10-dBm channel power. An acoustic-optical modulator (AOM) generates 1.375-μs time windows optimized for photoacoustic microscopy depth requirements. The subsequent time-division module, comprising high-reflectivity (>99 %) wavelength-matched FBGs and 150-meter delay fibers, creates 1.45-μs temporal intervals between channels. This configuration enables sequential signal detection through precise wavelength-to-time mapping.

The demodulation scheme exploits the fiber lasers' intrinsic birefringence to generate carrier frequencies through polarization beating. The beat frequencies for S1–S4 are 2.078 GHz, 1.956 GHz, 2.02 GHz, and 2.127 GHz respectively (Fig. 3c). Local oscillator mixing down-converts these frequencies to accommodate the data acquisition system's specifications (250 MHz sampling rate, 200 MHz bandwidth). Two key constraints are maintained: down-converted frequencies below 125 MHz and inter-channel beat frequency separations within 200 MHz. The resulting heterodyne signals (Fig. 3d) exhibit clear temporal separation between channels.

To validate the multiplexing capability, we conducted a proof-of-concept experiment where all four sensors were precisely aligned in parallel and placed in close proximity. This configuration ensures simultaneous detection of identical laser-induced ultrasound waves from a phantom absorber. The demodulated signals from individual sensors (Fig. 3e for all sensors and f-i for each) exhibit remarkably consistent SNRs of 31.7, 32.2, 30.9, and 31.8 dB, respectively. The minor variations between sensors are primarily attributed to inherent fabrication tolerances rather than multiplexing effects, as evidenced by the consistent noise floor across all channels. This uniform performance across all channels, despite their simultaneous operation in close proximity, demonstrates the effectiveness of our multiplexing strategy in maintaining channel independence and detection sensitivity. This result confirms the system's capability for crosstalk-free parallel detection.

## 3 Extended-FOV photoacoustic microscopy

**Fig. 4:**
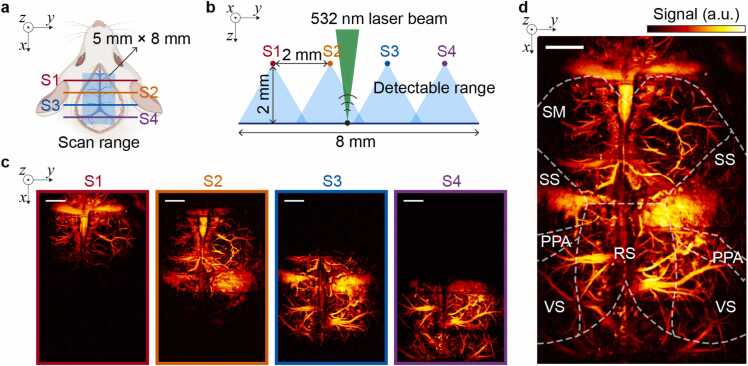
Multi-sensor array implementation for extended field-of-view photoacoustic microscopy (PAM). (a) Schematic illustration of the quadruple fiber-optic sensor array (S1–S4) configuration, covering a 5 mm × 8 mm scanning area. (b) Cross-sectional view of the *in vivo* imaging architecture demonstrating the optimized 2-mm inter-sensor spacing design for comprehensive cortical vasculature mapping. The 532-nm laser beam excitation and sensor detectable ranges are indicated. (c) Individual maximum amplitude projection images acquired from sensors S1–S4, demonstrating complementary spatial coverage and signal detection capabilities. (d) Integrated wide-field reconstruction through multi-sensor data fusion. Annotated anatomical regions include the somatomotor (SM), somatosensory (SS), retrosplenial (RS), visual (VS), and posterior parietal association (PPA) areas. The dashed lines delineate major functional boundaries. Scale bar: 1 mm.

Next, we deployed the multiplexed sensor array in large-field photoacoustic microscopy. The sensor array spans a 5 mm × 8 mm scanning area with optimized 2-mm inter-sensor spacing, ensuring effective overlap of uniform detection region for comprehensive cortical coverage (Fig. 4a, b). The imaging system integrates a 532-nm nanosecond pulsed laser (VPFL-G-HE-30, Spectra Physics) operating at 160 kHz for photoacoustic excitation, and a two-axis galvanometric scanner (GVS012, Thorlabs) coupled with a scanning lens (LSM03-VIS, Thorlabs) achieving ∼10 μm lateral resolution. The parallel-aligned sensor array, immersed in water for acoustic coupling, operates within a 6-μs total acquisition window (Fig. 3e). This temporal configuration aligns optimally with the 160 kHz laser repetition rate, while maintaining flexibility for potential speed enhancement through delay fiber optimization. In vivo imaging was conducted following institutional animal care protocols (GB/T 35892-2018, China). The preparation involved isoflurane anesthesia (1.5 % in medical air) and skull thinned for optimal acoustic transmission. Using 200 nJ pulse energy, we acquired images at 250 Hz B-scan rate with 640 × 1000 pixels resolution. Each sensor independently captured signals from its designated cortical region (Fig. 4c), while the integrated dataset enabled comprehensive visualization of the cortical vasculature.

To ensure optimal image quality, we implemented a systematic calibration and compensation strategy. Although our sensor array provides complete coverage of the entire cortical surface with each sensor's effective FOV (3 mm × 5 mm) exceeding the required imaging area, natural sensitivity variations exist due to the acoustic detection characteristics. We characterized these variations by imaging a uniform black tape target (Fig. 2e) and generated a compensation matrix. This compensation accounts for two factors: the distance-dependent signal attenuation between tissue and individual sensors, and the inherent sensitivity variations among sensors. The compensation matrix was then applied during image reconstruction to achieve uniform sensitivity distribution across the entire field of view, ensuring consistent image quality for both structural and functional imaging. The sensors were positioned with 2 mm spacing, creating partial overlap between adjacent detection regions to ensure complete coverage while maintaining high SNR (>30 dB) throughout the imaging field. During image reconstruction, signals from each sensor's optimal detection range (2 mm × 5 mm) were integrated. The composite reconstruction (Fig. 4d) achieved an 8 mm × 5 mm FOV, revealing detailed cerebrovascular networks across multiple functional regions including the somatomotor (SM), somatosensory (SS), retrosplenial (RS), visual (VS), and posterior parietal association (PPA) areas. Notably, the apparent signal variations at the image boundaries (particularly in S2-S4 regions) correspond to the natural anatomical limits of the cortical surface rather than detection limitations. Some minor blood spots observed in the images are attributed to the standard skull-thinning surgical procedure, a common occurrence in cranial window preparations, and not related to the imaging parameters. Through in vivo imaging, we found this multiplexed approach offering several unique advantages, including elimination of mechanical scanning artifacts through the stationary sensor array, and the uniform sensitivity across the entire field of view after calibration. While the current 2-mm inter-sensor spacing creates substantial overlap between adjacent sensors' detection regions, this deliberate design choice ensures consistent high-SNR signal acquisition across the entire imaging field, particularly important for maintaining image quality at the boundaries between sensor coverage areas. The modular nature of our sensor array design offers considerable flexibility in configuration with adjustable sensor spacing to accommodate various scanning requirements and anatomical regions, making it versatile for diverse biomedical applications.

## 4 Imaging of hemodynamic responses

**Fig. 5:**
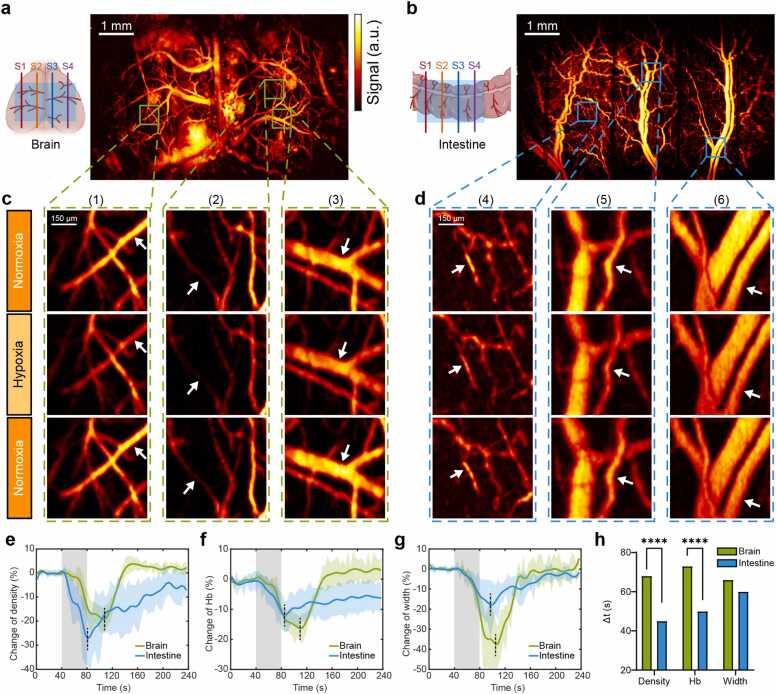
Comparative analysis of cerebral and intestinal hemodynamic responses to acute hypercapnia (50 % CO_2_ exposure) using wide-field photoacoustic microscopy. (a,b) Maximum amplitude projection images revealing vascular networks in the brain cortex (a) and intestinal tissue (b), with sensor array (S1–S4) coverage indicated in the anatomical diagrams. (c,d) Dynamic vascular responses in representative regions of interest (ROIs): magnified views from brain cortex (regions 1–3, c) and intestine (regions 4–6, d) captured during normoxia (baseline, 36 s), hypercapnia (108 s), and recovery phase (204 s). White arrows indicate vessels selected for quantitative analysis. (e-g) Temporal evolution of key vascular parameters demonstrating distinct response patterns between cortical and intestinal vasculature: vessel density (e), total hemoglobin concentration (f), and vessel diameter (g). (h) Comparative analysis of maximum response time (Δt) in vascular parameters between brain and intestinal tissue. Gray-shaded areas in e-g indicate CO2 exposure period (t = 40–80 s). n = 4. Data represent mean ± SD (e-g) and mean values (h). Statistical significance determined by two-way ANOVA (**** P < 0.0001).

We employed the extended-FOV photoacoustic microscopy to visualize tissue-specific vascular responses during hypercapnic challenge (supplementary video). The protocol comprised three phases: baseline normoxia (0–40 s), hypercapnic challenge (50 % CO_2_, 40–80 s), and recovery (80–240 s). Physiological parameters were maintained at 1.5 % isoflurane anesthesia and 37 °C body temperature, with subject either stereotaxically fixed (brain imaging) or positioned supine post-laparotomy (intestinal imaging). The extended-FOV system enabled simultaneous monitoring of multiple vascular beds through the four-sensor array configuration, as illustrated in Fig. 5a,b. High-resolution maximum amplitude projection images revealed distinct vascular networks in both brain cortex and intestinal tissue. Time-series analysis of selected regions of interest (ROIs) demonstrated dynamic vascular responses across three critical timepoints: baseline (36 s), peak response (108 s), and recovery (204 s). Representative regions from brain cortex (Fig. 5c) and intestine (Fig. 5d) were analyzed, with specific vessels marked. Vascular dynamics were quantified through three parameters: vessel density (vessel-to-tissue area ratio), hemoglobin concentration (Hb, mean signal intensity), and vessel width (FWHM from Gaussian fitting of 5-fold interpolated vessel profiles).

Quantitative assessment of vascular dynamics focused on three key parameters: vessel density (vessel-to-tissue area ratio), hemoglobin concentration (Hb, mean signal intensity), and vessel width (FWHM from Gaussian fitting of 5-fold interpolated vessel profiles). Time-series analysis of selected regions of interest (ROIs) from brain cortex (Fig. 5c) and intestine (Fig. 5d) demonstrated significant tissue-specific responses (p < 0.0001) across three critical timepoints: baseline (36 s), peak response (108 s), and recovery (204 s). Statistical analysis revealed distinct response patterns: intestinal tissue exhibited greater reduction in vessel density (27.3 %) compared to brain cortex (19.2 %) during hypercapnia (Fig. 5e), while hemoglobin concentration showed comparable reductions in both tissues (brain: 16.4 %, intestine: 12.1 %; Fig. 5f). Brain vessels demonstrated more pronounced constriction (37.6 % reduction) versus intestinal vessels (17.8 % reduction; Fig. 5g). Notably, temporal analysis (Fig. 5h) revealed significantly delayed maximum response time in cerebral vessels density compared to intestinal vessels density (Δt = 68 s vs 45 s, p < 0.0001) and in cerebral Hb compared to intestinal Hb (Δt = 73 s vs 50 s, p < 0.0001). These temporal dynamics during CO_2_ exposure (gray-shaded regions, t = 40–80 s) suggest distinct autoregulatory mechanisms between cerebral and intestinal vasculature, with cerebral circulation exhibiting more robust but delayed responses, potentially reflecting a protective mechanism for maintaining stable cerebral blood flow. Our multiplexed photoacoustic microscopy approach successfully captured these nuanced spatiotemporal variations in vascular dynamics, providing quantitative insights into tissue-specific vascular regulation mechanisms during physiological challenges.

## 5 Discussion and conclusion

Our WTDM fiber-optic sensor array introduces an alternative approach to parallel optical ultrasound detection and wide-field photoacoustic imaging. The hybrid wavelength-time division multiplexing strategy effectively resolves the long-standing sensitivity-multiplexing trade-off in multi-channel detection systems. By implementing temporal separation, we eliminate inter-channel crosstalk—a persistent challenge in conventional WDM approaches—while maintaining uniform signal-to-noise ratios across all channels. In comparison, as shown in Table 1, although the optical resonators (phase-shifted gratings or microring resonators) interrogated with optical frequency combs [51], [52] achieved multiplexing capability of up to 15 sensors per channel, its sensitivity, in terms of NEP degraded. Moreover, our single-photodetector architecture, combined with strategically overlapped detection fields, achieves comprehensive large-area coverage while significantly reducing system complexity and cost.

**Table 1:** Comparison of multiplexed optical ultrasound sensors.

**Type**	**Noise-equivalent pressure (NEP)**	**Bandwidth**	**Sensor number per channel (photodetector and acquisition channel)**	**In vivo imaging capability**
Phase-shifted gratings with pulse interferometry [51]	100 Pa	16 MHz	1	no
Photothermally tunable Fabry–Perot sensors [53]	120 Pa	20 MHz	1	no
Two-dimensional Fabry–Perot sensor array [55]	287–456 Pa	18.3 MHz	1	no
Micro-ring resonators with digital optical frequency comb [52]	36.9 Pa	20 MHz	15	Sensor-scanning PACT
Planar Fabry–Perot sensor with multiple probe lights [54]	250 Pa	35 MHz	1	PACT
Fiber laser sensor (this work)	8 Pa	16 MHz	4	PAM

The WTDM system architecture demonstrates inherent scalability in both sensor numbers and layouts. While our current 4-sensor implementation effectively serves PAM applications, the architecture provides a solid foundation for array expansion through additional DWDM channels. The primary technical considerations for scaling include available wavelength channels, photodetector bandwidth, saturation power, and optimization of time-delay management. Notably, our demonstration of uniform SNR maintenance and effective cross-talk suppression across channels suggests promising potential for larger arrays. This scalability enables large FOV imaging with sub-linear cost scaling, as our WTDM approach maintains the same optical path and processing architecture regardless of sensor count.

Furthermore, the flexible layout of fiber-optic sensors enables customized array configurations for specialized applications. This flexibility is particularly valuable for in vivo imaging scenarios where adapting to complex anatomical structures is crucial. For instance, sensors can be arranged to conform to curved surfaces or strategically positioned for simultaneous monitoring of multiple regions of interest. This adaptability, combined with our sensors' high sensitivity and temporal resolution, opens new possibilities for studying inter-organ interactions, such as brain-gut axis dynamics. By simultaneously monitoring both brain tissue and intestinal regions with precisely positioned sensor arrays, researchers could gain unprecedented insights into the temporal correlation of physiological events across multiple organs. Such capability would be particularly valuable for investigating complex physiological phenomena like neuroimmune interactions and systemic responses to various stimuli, while maintaining the high-fidelity imaging performance demonstrated in our current implementation.

Several technical challenges currently limit system performance. Random fiber birefringence affects microwave carrier frequencies, leading to variable beat frequencies between sensors and limited multiplexing capacity. This can be addressed through implementation of specialty fiber designs with controlled birefringence properties and development of stress-induced birefringence control mechanisms for precise frequency management. Time-division multiplexing introduces trade-offs between channel count, sampling rate, and imaging depth, currently limiting A-line rates to 166 kHz. These temporal constraints can be mitigated through advanced hybrid frequency-time domain multiplexing schemes and optimized delay fiber configurations. Additionally, the current balance between imaging speed, FOV, and resolution results in some missing image details, which can be enhanced through advanced upsampling algorithms and resolution enhancement techniques for small vessel visualization[16].

In conclusion, our WTDM fiber-optic ultrasonic sensing platform represents a significant advancement in parallel ultrasound detection and high-speed photoacoustic imaging. By integrating wavelength-division multiplexing with temporal encoding, our system achieves optimal sensitivity across all channels while maintaining signal integrity through a single-photodetector architecture. The four-element sensor array implementation has enabled a four-fold FOV expansion to 5 × 8 mm² while preserving high temporal resolution and microscopic spatial resolution in optical scanning method. This capability has been successfully demonstrated in comparative studies of cerebral and intestinal hemodynamics, revealing distinct temporal patterns in vascular responses during hypoxic challenges. While acknowledging current technical limitations, our proposed solutions and the platform's demonstrated performance establish a promising foundation for comprehensive biomedical imaging applications, from cortical examination to minimally invasive endoscopic diagnostics. The system's ability to maintain high sensitivity while enabling expanded field-of-view imaging, combined with its clear pathway for future improvements, positions this technology as a valuable tool for advanced biomedical research and clinical applications.

## CRediT authorship contribution statement

**Yinyan Lai:** Resources. **Hao-Cheng Tang:** Resources. **Bai-Ou Guan:** Funding acquisition. **Jie Huang:** Investigation. **Xiaoxuan Zhong:** Writing – review & editing, Visualization, Software, Conceptualization. **Wei Li:** Writing – original draft, Visualization, Software, Investigation. **Long Jin:** Writing – review & editing, Supervision, Funding acquisition, Conceptualization. **Linghao Cheng:** Supervision. **Yizhi Liang:** Methodology. **Xue Bai:** Methodology.

## Declaration of Competing Interest

The authors declare that they have no known competing financial interests or personal relationships that could have appeared to influence the work reported in this paper.

## Data Availability

Data will be made available on request.
